# Dr. Eugene Edward Martin

**Published:** 1904-12

**Authors:** 


### OBITUARY.

Dr. Eugene Edward Martin, of Buffalo, died at his home, 753 Michigan street, November 23, 1904, aged 37 years. Dr. Martin graduated at Niagara University in 1888, since which time he has been engaged in the practice of medicine in this city.

